# When gender equality initiatives risk doing more harm than good

**DOI:** 10.1016/j.eclinm.2020.100330

**Published:** 2020-04-30

**Authors:** Catherine Moughalian, Susanne Täuber

**Affiliations:** a University Medical Centre Groningen (UMCG), the Netherlands; b Department of Human Resource Management & Organizational Behavior, University of Groningen, Nettelbosje 2, 9712 TS, Groningen, the Netherlands.

The recent issue on gender equality in *EClinicalMedicine* underscores the importance of institutional action to overcome gender discrimination. But the progress resulting from gender equality initiatives is disappointing [1], suggesting a potential blind spot concerning intersectionality and institutional responses to complaints.

Gender is a complex multidimensional phenomenon [2]. Inequality regimes within institutions are based on the intersections of gender, class, ethnicity, citizenship, sexuality, and physical ability. Women's experiences and opportunities within organizations depend on the interplay of all these categories. When organizations are more concerned with their reputation than with changing existing inequality regimes, gender equality initiatives create an illusion of institutional commitment to diversity and inclusion that masks persistent abuses of power. Sexual harassment is pervasive in science and academic medicine [3], but women speaking up against it are silenced and threatened, denied their experiences, or pressured into leaving their positions.

Inequality regimes are deeply ingrained in our universities and medical schools. In the absence of comprehensive institutional action, gender equality initiatives risk doing more harm than good. Research shows that those in power frequently compensate for the ostensibly “unfair” advantages women get from such initiatives, for instance by withholding resources and information [4]. An institutional culture of accountability can prevent such “undoing” of gender equality [5]. Those in privileged positions need to recognize their power and stand in solidarity with women affected by harassment or discrimination. Our thinking needs to shift from shallow policies and gender mainstreaming programs to an explicitly political, feminist, and intersectional approach that seeks to unhinge heteropatriarchal power structures.
